# Two to Tango: Kidney-Lung Interaction in Acute Kidney Injury and Acute Respiratory Distress Syndrome

**DOI:** 10.3389/fped.2021.744110

**Published:** 2021-10-18

**Authors:** Joseph Alge, Kristin Dolan, Joseph Angelo, Sameer Thadani, Manpreet Virk, Ayse Akcan Arikan

**Affiliations:** ^1^Division of Nephrology, Department of Pediatrics, Baylor College of Medicine, Texas Children's Hospital, Houston, TX, United States; ^2^Division of Critical Care Medicine, Department of Pediatrics, Baylor College of Medicine, Texas Children's Hospital, Houston, TX, United States

**Keywords:** AKI, ARDS, lung, PARDS, fluid overload

## Abstract

Acute Kidney Injury (AKI) is an independent risk factor for mortality in hospitalized patients. AKI syndrome leads to fluid overload, electrolyte and acid-base disturbances, immunoparalysis, and propagates multiple organ dysfunction through organ “crosstalk”. Preclinical models suggest AKI causes acute lung injury (ALI), and conversely, mechanical ventilation and ALI cause AKI. In the clinical setting, respiratory complications are a key driver of increased mortality in patients with AKI, highlighting the bidirectional relationship. This article highlights the challenging and complex interactions between the lung and kidney in critically ill patients with AKI and acute respiratory distress syndrome (ARDS) and global implications of AKI. We discuss disease-specific molecular mediators and inflammatory pathways involved in organ crosstalk in the AKI-ARDS construct, and highlight the reciprocal hemodynamic effects of elevated pulmonary vascular resistance and central venous pressure (CVP) leading to renal hypoperfusion and pulmonary edema associated with fluid overload and increased right ventricular afterload. Finally, we discuss the notion of different ARDS “phenotypes” and the response to fluid overload, suggesting differential organ crosstalk in specific pathological states. While the directionality of effect remains challenging to distinguish at the bedside due to lag in diagnosis with conventional renal function markers and lack of tangible damage markers, this review provides a paradigm for understanding kidney-lung interactions in the critically ill patient.

## Introduction

Acute respiratory distress syndrome (ARDS) is a life-threatening condition and a leading cause of mortality in critically ill patients causing nearly 200,000 deaths in the United States each year (1). The development of acute kidney injury (AKI) is common in patients with ARDS. AKI significantly adds to the morbidity and mortality of patients with ARDS. In the ARDSNet trial, patients with AKI and ARDS had almost twice the mortality rate than those with ARDS alone (2).

The implications of AKI are broad and include fluid overload, electrolyte abnormalities, immunoparalysis, and multiple organ dysfunction through organ crosstalk. Respiratory complications are a key marker of increased mortality in patients with AKI, which points to a biologically plausible mechanistic link between AKI and Acute Lung Injury (ALI). Animal models show AKI causes ALI (3). Conversely, mechanical ventilation and ALI can lead to AKI, further supporting a bidirectional relationship (4).

**Figure 1:**
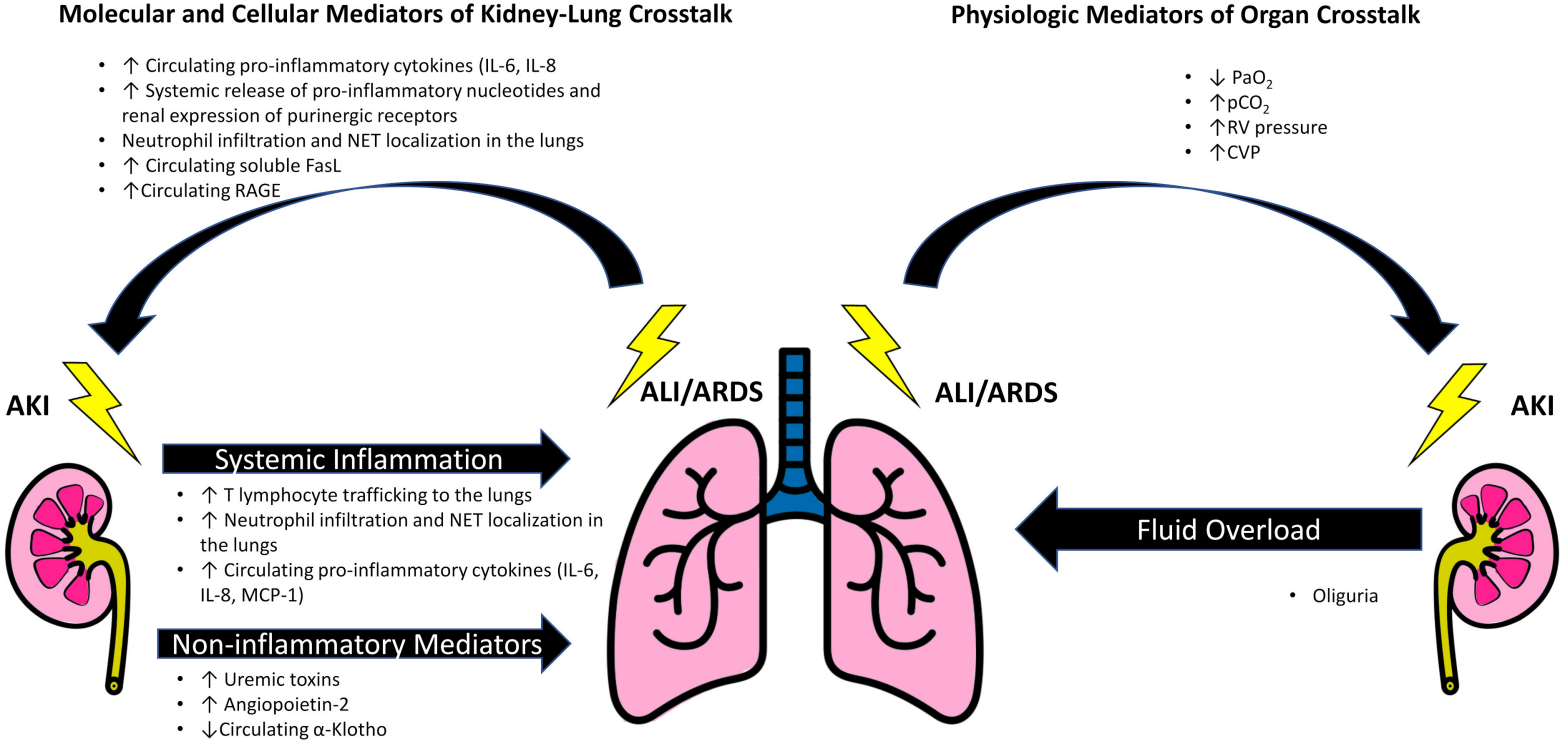
Schematic of mediators of kidney-lung crosstalk in AKI and ARDS. Acute kidney injury propagates acute lung injury and contributes to the development of acute respiratory distress syndrome (ARDS) by generation of inflammatory cytokines, such as IL-6, increased trafficking of T lymphocytes and neutrophils to the lungs. Additionally, AKI can contribute to the development of pulmonary edema and ARDS due to the combination of fluid overload, changes in expression of pulmonary epithelial Na channels and aquaporin-5 mediated by uremic toxins, and angiopoietin-2 induced vascular leak. A reduction in circulating α-Klotho after AKI increases the lungs' susceptibility to oxidative damage. Factors that promote renal injury after ALI/ARDS include a systemic inflammatory response associated with mechanical ventilation. Key lung mediated factors include IL-6, nucleotides, and soluble receptor for advanced glycation end products (RAGE). Increased circulating FasL induces renal tubular epithelial cell apoptosis. Additionally, the ALI/ARDS may reduce renal blood flow through the effects of hypoxia and hypercapnia on intrarenal vasculature, as well by the direct action of elevated right ventricular and central venous pressures in opposition to renal perfusion.

There are many challenging and complex interactions between the lung and kidney in critically ill patients with AKI and ARDS (Figure 1). These include disease specific molecular mediators and inflammatory pathways unique to ARDS and AKI that are involved in organ crosstalk. Additionally, there are patient characteristics such as hemodynamics, comorbidities, and host factors such as genetic susceptibility in AKI that can lead to worsening ARDS. Recently, different ARDS “phenotypes” have been proposed via utilization of unsupervised clustering with or without the combination of unique biomarkers (1, 5). An intriguing aspect of these models has been demonstration of a differential response to fluid, which might suggest discrete mechanisms of organ crosstalk in specific pathological states. Finally, there are likely reciprocal hemodynamic effects of elevated pulmonary vascular resistance and central venous pressure (CVP) leading to renal hypoperfusion and pulmonary edema associated with fluid overload, which could lead to increased right ventricular afterload. Each of these interactions contribute to the ARDS-AKI construct and highlight how the implications of AKI go beyond just the kidney. The directionality of effect remains challenging to distinguish at bedside due to a lag in diagnosis with conventional renal function markers and lack of tangible damage markers in both AKI and ARDS. Thus, it is important to have an in depth understanding of this relationship. This review provides a paradigm for understanding the kidney-lung interactions in critically ill patients.

## Kidney Mediated Lung Injury

Elucidation of the pathophysiology of AKI has revealed a complex, multisystem disorder with clinically important implications for patients with AKI. Important among these is the relationship between AKI, acute lung injury (ALI) and acute respiratory distress syndrome (ARDS). Data from studies in multiple clinical settings and animal models support a dynamic interplay between the kidneys and lungs with each set of organs having the potential to influence and disrupt the structure and function of the other (Table 1). In the setting of AKI, injury to the kidneys can have distant effects on the lungs through both cellular and circulating biochemical mediators. These warrant further investigation as biomarkers and potential therapeutic targets and include both inflammatory and non-inflammatory mediators.

**Table 1:** Selected publications highlighting kidney mediated lung injury.

**Authors**	**Key findings**
**Inflammatory mediators of lung injury**	
Lie et al. (6)	Renal ischemia-reperfusion injury is associated pulmonary epithelial cell apoptosis mediated by increased trafficking of CD8+ T lymphocytes to the lungs
Nakazawa et al. (7)	Renal ischemia-reperfusion injury leads to increased neutrophil extracellular trap formation in the lungs and is associated with pulmonary epithelial cell apoptosis
Klein et al. (3)Ahuja et al. (8)	IL-6 deletion or blockade attenuate lung inflammation and injury after renal ischemia-reperfusion injury (mouse model)
**Non-inflammatory mediators of lung injury**	
Rabb et al. (9)	Renal ischemia-reperfusion injury and bilateral nephrectomy are associated with pulmonary edema and altered expression of the pulmonary epithelial Na channel and aquaporin-5
Yabuuchi et al. (10)	The uremic toxin indoxyl sulfate accumulates in the lungs after renal ischemia-reperfusion injury and induces altered pulmonary expression of aquaporin-5
Hsia et al. (11)	Supplementation with alpha Klotho, which is normally decreased after AKI, attenuates lung damage after renal ischemia-reperfusion injury
de Vries et al. (12)	Renal ischemia-reperfusion injury releases systemic angiopoietin-2
Zinter et al. (13)	Elevated plasma angiopoietin-2 concentration is associated with increased mortality in children with ARDS
Alobaidi et al. (14)	Fluid overload is associated with mortality in critically ill children

### Inflammatory Mediators of ARDS in the Setting of AKI

#### Cellular Inflammatory Mediators

Several populations of immune cells, including T-cells, neutrophils and macrophages, have been shown to mediate lung injury in the setting of AKI. Evidence for the involvement of T-cells in distant organ effects in the setting of AKI comes initially from investigation of the actions of T cells following organ specific injury. For example, in a mouse model of ischemia-reperfusion AKI, T-cell trafficking to the injured kidney has been shown to occur within 1 hour of the time of insult and to result in the local release of proinflammatory mediators (15). Furthermore, a major target of this T-cell recruitment and proinflammatory activation are endothelial cells, resulting in disruption of the renal microvascular barrier and an increase in renal microvascular permeability (15, 16). Extending these findings to potential mechanisms of T-cell mediated lung injury in AKI, Lie et al. examined T-cell trafficking and activation in a mouse model of ischemic AKI and found that, compared to sham controls, the number of T-cells, specifically CD8+ T-cells, was significantly increased in the lungs of mice with ischemic AKI (6). In addition, markers of T-cell activation were also increased (6). Together, these findings suggest that in AKI, T-cells are not only targeted to the lungs but also that these T-cells display markers of activation at the time of infiltration into the lungs. This study also showed that T-cells mediate pulmonary cell apoptosis, as evidenced by an increase in caspase-3 activity in the pulmonary tissue of mice following ischemic AKI, and that this pulmonary apoptosis results in disruption of the pulmonary microvascular barrier shown by increased bronchoalveolar lavage (BAL) total protein (6). Interestingly, this apoptotic effect was not present in T-cell depleted mice and was restored by adoptive transfer and reconstitution of T cells in the T-cell deficient mice (6). In aggregate, these animal model findings highlight the influence of T-cells not only in modulating injury to the kidney in AKI but also show the impact that T-cell trafficking, and activation can have on the lungs in the setting of AKI.

Similar to T-cells, both neutrophils and macrophages have been shown to be involved in the mediation of organ specific injury in both the kidney and the lung. In several models of AKI, including ischemia and nephrotoxin associated AKI, resident renal macrophages are involved in the initial response to injury and are targeted to the sites of injury through the release of damage associated molecular pattern (DAMP) molecules and hypoxia inducible factors (HIFs) from injured renal tubular epithelial cells (17, 18). This process occurs relatively quickly during the first 24 hours following injury (19), and subsequently, these recruited macrophages perpetuate the inflammatory response by recruiting other leukocytes including bone marrow derived macrophages, neutrophils, and lymphocytes through the secretion of proinflammatory signal molecules, such as tumor necrosis factor-alpha (TNF-α) (20, 21). The next steps in the macrophage response to tissue injury in AKI involve the regulation of inflammation and a shift in macrophage subtype from M1 macrophages that promote the initial rapid inflammatory response to the M2 subtype which move towards tissue repair (22).

Similar macrophage and neutrophil driven processes occur in ARDS, and highlight the importance of the balance between initial rapid pro-inflammatory phase, and the subsequent regulation of inflammation and shift to a more reparative milieu. One important neutrophil related intersection of AKI and ALI could be at the formation of neutrophil extracellular traps (NETs). NET formation is a unique form of bacterial killing and is important to the normal response to microbial infection, but it also has been shown to occur at sites of sterile inflammation including the development of AKI and ALI/ARDS (23, 24). Interestingly, in a mouse model of ischemic AKI, NET formation was not only shown to amplify kidney damage, but NET levels and apoptosis were also increased in the lungs, suggesting a role for NETs as a circulating factor mediating ALI/ARDS following AKI (7). While an appropriately targeted cellular response to tissue injury is a critically important adaptive response, the above noted data support the idea that a dysregulated and exaggerated inflammatory response can potentially worsen not only local tissue damage at the initial site of injury, but also might cause injury at distant sites in other organs.

#### Soluble Inflammatory Mediators

In addition to cellular mediators of tissue injury, several soluble factors have been reported to trigger ARDS in the setting of AKI. Similar to the cell driven inflammatory responses outlined above, these inflammatory molecules have been shown to mediate damage not only locally but also have been implicated in distant organ effects including causing ALI in the setting of AKI (25).

Important among the molecular mediators of lung injury associated with AKI is interleukin-6 (IL-6). Clinically, serum IL-6 levels have been shown to increase in patients following AKI, and also have been associated with prolonged mechanical ventilation in the setting of AKI (26). Regarding the pulmonary effects of IL-6 in AKI, a study by Klein et al. showed increased pulmonary inflammation, increased neutrophil recruitment to the lungs, and increased pulmonary capillary leak using a mouse model of renal ischemic injury and also in a bilateral nephrectomy model (3). In this model, IL-6 deficient mice and mice treated with IL-6 antibody showed an attenuated lung inflammatory response following AKI despite similar levels of renal dysfunction (3). As a corollary to this, intravenous injection of IL-6 to IL-6 deficient mice has been shown to restore lung inflammation implicating circulating, and not local pulmonary, IL-6 in driving pulmonary inflammation following AKI (8). Intriguingly, inhaled IL-6 might actually provide a protective effect for the lungs compared with circulating IL-6, highlighting circulating IL-6 as a mediator of distant lung injury after AKI (27).

Similar to IL-6, interleukin-8 (IL-8) has been studied with regard to its involvement in tissue specific injury in AKI and ALI as well as in AKI mediated lung injury. There is evidence that IL-8 is increased in the serum of patients with AKI following cardiac surgery, and that this elevation in IL-8 is associated with prolonged mechanical ventilation (26). Also similar to IL-6, mice deficient in CXCL1, the mouse functional analog of human IL-8, or treated with anti CXCL1 antibodies, have been shown to be protected from AKI associated lung injury (8, 28). In this way, both circulating IL-6 and IL-8 are shown to play a strong role in generating and perpetuating injury to the lungs following AKI.

In contrast to IL-6 and IL-8, other circulating cytokines appear to play a more anti-inflammatory role in modulating lung injury following AKI. In a mouse model of AKI using bilateral nephrectomy, treatment with IL-10 was shown to decrease pulmonary edema, neutrophil infiltration and BAL fluid protein, indicating a protective effect of IL-10 on the lungs of these experimental animals with AKI (29). Conflicting data exist on the impact of extracorporeal blood purification and its impact on mitigation of these mechanisms of kidney and lung injury, a complete review of this topic is beyond the scope of this manuscript.

### Non-inflammatory Mediators of ARDS in the Setting of AKI

#### Uremic Lung: Resurrecting an Old Idea

The link between AKI and subsequent ARDS was first recognized in the mid-20th century when clinicians coined the term “uremic lung” (30). While this antiquated nomenclature has fallen into disuse, it was in fact quite prescient because it encapsulates the role of uremic toxins in kidney lung crosstalk. Uremic toxins such as indoxyl sulfate and p-cresyl sulfate contribute to the development of ARDS via their pro-inflammatory effects and through direct effects on pulmonary gene expression leading to dysregulated fluid handling in the lungs. While induction of pro-inflammatory genes in the lung and histologic evidence of pulmonary inflammation is more severe after renal ischemia reperfusion injury, mice that undergo bilateral nephrectomy also demonstrate increased pulmonary expression of *TNFa, IL-6*, and *CXLCL-1* (3, 31, 32). In fact, lung injury in nephrectomized mice is markedly attenuated in IL-6 knockouts or treatment with an IL-6 neutralizing antibody (3). However, the proinflammatory effects of bilateral nephrectomy may not be solely explained by reduced clearance of IL-6, since more recent studies have shown that indoxyl sulfate is a potent inducer of IL-6 expression through the aryl hydrocarbon receptor and NFκB pathways (33–35). In addition to their proinflammatory effects, uremic toxins accumulate in the lungs and pleural fluid where they contribute to the development of non-cardiogenic pulmonary edema by dysregulated Na and water clearance via downregulated expression of epithelial Na channels, the Na,K ATPase, and aquaporins-1 and -5 (9, 10, 36, 37). Finally, uremic toxins are well-known inducers of endothelial dysfunction, which is mediated by increased reactive oxygen species production and downregulation of antioxidant genes such as *Nrf-2*, α*-Klotho, and Heme oxygenase-1* and have been implicated as proinflammatory mediators of endothelial dysfunction (38). While this has not been demonstrated specifically in the pulmonary vasculature, it is biologically plausible that endothelial dysfunction caused by uremic toxins contributes to pulmonary vascular leak. Therefore, uremic toxins can contribute to the development of ARDS by several mechanisms, and future studies should investigate the ability of these biomarkers to identify patients with AKI who are at risk of developing pulmonary complications.

#### α-Klotho

α-Klotho has recently emerged as an important mediator of kidney-lung crosstalk in the setting of AKI. Initially identified as an anti-aging gene, disruption of α-Klotho expression in mice leads to early death due to progressive multi-organ failure that resembles accelerated aging and is associated with emphysematous changes in the lung (39). However, α-Klotho is not expressed in the lung, its expression is restricted predominantly to the kidney and parathyroid glands, where it functions as the co-receptor for FGF-23, a hormone that is a critical regulator of phosphate metabolism (40, 41). α-Klotho exists as both a type-I single pass transmembrane protein and a soluble form, which is generated either by alternative splicing or proteolytic cleavage of the ectodomain of the membrane bound form (42, 43). Soluble α-Klotho has pleiotropic cytoprotective effects and is predominantly derived from the kidney (11, 44, 45). Of note, AKI leads to a precipitous decrease in soluble α-Klotho, and in a rodent model of ARDS after AKI, repletion of α-Klotho provided protection of subsequent oxidative lung injury through upregulation of downstream antioxidant effectors of the Nrf2 pathway (46–49). Therefore, α-Klotho has been proposed both as a biomarker for predicting the development of AKI-induced ARDS and as a potential therapeutic target.

#### Angiopoietin-2 – Endothelial Damage Marker

Angiopoietin-2 (Ang-2) has been identified as a mediator of vascular permeability in patients with sepsis and ARDS, and it could play a pivotal role in kidney-lung crosstalk (50–53). Ang-2 is stored in Weibel-Palade bodies in endothelial cells and is released in response to endothelial activation (54). It acts in an autocrine manner as an antagonist for the Tie-2 receptor, which is predominantly expressed by endothelial cells to promote vascular leakage, and it is opposed by the agonistic action of angiopoietin-1 (55). As a biomarker, polymorphisms in the *Ang-2* gene have been linked to increased risk of ARDS, and higher serum Ang-2 levels correlate with impaired oxygenation and an increased risk of mortality in patients with ARDS (13, 56, 57). The angiopoietin/Tie-2 signaling axis plays an important role in vascular development during nephrogenesis, and a study of renal transplant recipients demonstrated that renal ischemia/reperfusion injury leads to rapid release of Ang-2, suggesting that Ang-2 could contribute to the development of ARDS in patients with AKI (12, 58, 59).

## Lung Mediated Kidney Injury

Available data support the concept of distant organ effects on the lungs in AKI caused by a complex interplay between multiple cellular and molecular modulators of the immune system, which can initiate and perpetuate damage to the lungs. This is all under the assumption that the initial site of injury is the kidneys, and it is important to recognize the bidirectionality of kidney-lung crosstalk. Despite the high incidence of AKI in patients with ARDS, the impact of ALI on this ARDS-AKI construct and the effects of mechanical ventilation on the kidneys are not well understood (1). The leading hypotheses of lung mediated AKI include the direct effects of hypoxemia and hypercarbia on renal blood flow and renal cell injury, the systemic inflammatory response, and other molecular mediators causing direct renal cell damage (Table 2).

**Table 2:** Selected publications highlighting lung mediated renal injury.

**Authors**	**Key findings**
**Effects of hypoxemia and hypercapnea on renal perfusion**	
Darmon et al. (60)	Hypoxemia reduces renal perfusion, increases renal resistive index
Sharkey et al. (61)	Hypoxia and hypercapnia increase renal resistive index and reduce renal perfusion independent of the sympathetic nervous system
**Renal hemodynamic effects of ARDS**	
Ottolina et al. (62)	Higher PEEP is associated with increased risk of AKI in patients with COVID-19
**Inflammatory mediators of kidney injury**	
Douillet et al. (63)	Mechanical ventilation induces release of nucleotides from pulmonary tissue and altered expression of purinoreceptors in the kidneys
Imai et al. (4)	Injurious mechanical ventilation strategies increase systemic levels of inflammatory cytokines, chemokines, and soluble FasL is associated with proximal tubule epithelial cell apoptosis
Ranieri et al. (64)	Effect of mechanical ventilation on systemic and local production of inflammatory cytokines
Parsons et al. (65)	Lung-protective ventilation strategy reduces systemic levels of proinflammatory cytokines
Calfee et al. (66)	Acute lung injury is associated with increased plasma concentration of receptor for advanced glycation end products (RAGE)
**Subphenotypes of ARDS**	
Calfee et al. (67)	Identification of ARDS subphenotypes using molecular phenotyping
Famous et al. (5)	Differential response of ARDS subphenotypes respond to fluid management

### Hypoxemia, Hypercapnia, and Tissue Hypoxia

ALI and ARDS often lead to hypoxemia and hypercapnia, both of which affect renal blood flow and can lead to direct renal ischemia and subsequent injury. While the hallmark of ARDS is hypoxemic respiratory failure, hypercapnia can result from either the primary disease process, lung protective strategies limiting ventilation, or frequently, a combination of the two. Specifically, the current recommendations for treatment of ARDS include targeting lung protective strategies with lower goals for oxygen levels (both partial pressure of arterial oxygen and systemic saturations) and permissive hypercapnia (60). Both hypoxemia and hypercarbia have significant effects on renal blood flow and potentially lead to the development and progression of AKI. The presence of severe hypoxemia (Pa02 < 40) leads to decreased renal blood flow and renal dysfunction (60). There are conflicting reports regarding moderate or mild hypoxemia on renal blood flow. Some studies suggest acute hypoxemia, even at mildly low systemic oxygen saturation levels of 88%, causes an acute decrease in renal blood flow while others suggest there is a kidney-lung protective strategy with mild hypoxemia with an increase in urine output, called the hypoxemic diuretic response (68). The mechanisms of hypoxemic changes in renal vascular tone and blood flow are largely unknown but thought to be related to activation and/or inactivation of nitric oxide, angiotensin II, endothelin, bradykinin, and the sympathetic reflex (61, 68, 69). The exact threshold of hypoxemia that is no longer “protective” and, instead, becomes injurious to the renal tissue bed requires further delineation. Hypercarbia, in turn, leads to decreased renal blood flow and perhaps has a stronger impact than hypoxemia, especially in the acute setting (60, 69). The arterial partial pressure of carbon dioxide is inversely related to renal blood flow in animal models and in subjects with normal respiratory physiology, acute respiratory failure, and chronic obstructive pulmonary disease. Hypercapnia directly causes renal vasoconstriction and systemic vasodilation inducing the release of noradrenaline and the activation of the renin-angiotensin-aldosterone system which also contributes to renal vascular tone (69). Decreased renal blood flow, in turn, leads to renal hypoxia, and apoptosis and necrosis with resultant vascular and tubular damage culminating in acute tubular injury (70). Highly metabolically active and energy dependent proximal tubular cells are most sensitive to hypoxic injury as the medulla becomes far more hypoxic than the renal cortex in states of decreased renal blood flow (71).

### Inflammatory Mediators of Kidney Damage in the Setting of ARDS

An additional suggested mechanism of lung-kidney crosstalk involves extracellular nucleotides released by injured pulmonary epithelial cells. Nucleotides are molecules that modulate numerous functions including vascular tone, apoptosis, membranous ion conductance, and trans alveolar fluid regulation in the lungs. Nucleotides are released by pulmonary epithelial cells in response to physical stimuli including shear stress during mechanical ventilation. In addition, nucleotides induce the synthesis and release of cytokines, specifically IL-6, which is a proinflammatory mediator described in the pathogenesis of AKI and the generation of renal tubular injury (72, 73). Douillet et al. demonstrated that mechanical ventilation alters the nucleotide and purinoreceptor expression in the kidney, even in the presence of protective mechanical ventilation strategies, suggesting ongoing lung-kidney cross talk in the setting of lung injury and mechanical ventilation (63, 74).

Renal cell injury has also been suggested by Imai et al., who compared injurious and non-injurious ventilator strategies (4). Injurious ventilator strategies led to the production of inflammatory cytokines and chemokines IL-8, MCP-1, and GRO, all of which have been implicated in the pathophysiology of AKI (4, 75). Additionally, proximal tubular cell apoptosis was observed *in vitro* with elevated blood urea nitrogen and creatinine levels as well as *in vivo* in rabbit models with injurious ventilator strategies; hypothesized to be secondary to soluble Fas ligand (sFasL), an important mediator of renal cell injury via apoptosis (4). Lastly, nitric oxide, a vasodilator that has been shown to have systemic and renal cytotoxic effects, has been implicated in ARDS-AKI construct. Choi et al. showed that injurious mechanical ventilation induced nitric oxide synthase (NOS) expression in both the lung and kidney, causing release of vascular endothelial growth factor causing increased vascular permeability and cytokine release (76).

### Mechanical Ventilation as a Pro-inflammatory state

In addition to direct effects of hypoxemia, tissue hypoxia, and hypercapnia, lung injury involves multiple cytokines, chemokine, and pro-inflammatory pathways leading to kidney specific injury and the development of AKI. Mechanical ventilation, especially when higher tidal volumes and mean airway pressure are used and lung protective principles are not applied, propagates lung injury through atelectotrauma and sheer stress and contributes significantly to the release of damage associated molecular patterns (DAMPs), and various cytokines, and chemokines (77). In fact, inflammatory cytokines in patients ventilated with lung protective strategy were significantly decreased compared to controls who received standard mechanical ventilation in randomized control trials, with a noted reduction in bronchoalveolar concentrations of polymorphonuclear cells, TNF-α, IL-1β, soluble TNF-α receptor, and IL-8 (64). Plasma and bronchoalveolar levels of IL-6, IL-8, soluble TNF-α receptor, and IL-1 receptor antagonist were also decreased, all of which have been implicated in the pathophysiology of AKI (64, 65). It is reasonable to surmise that even in the absence of a primary pulmonary pathology, injurious ventilation strategies could contribute to the organ crosstalk, leading to remote organ injury manifested as AKI (65). Receptor for Advanced Glycation End Products (RAGE) is considered an alveolar epithelial injury marker and levels can increase in the serum within an hour after a recruitment maneuver, which is typically characterized by brief but sustained increase in airway pressure in order to recruit collapsed segments of injured lung (78). Certain subgroups of adult ARDS patients have higher RAGE levels, suggesting more epithelial damage (79, 80). Soluble forms of these mediators spill over into circulation and are measurable in peripheral blood, an example that many other DAMPs contribute to remote signaling and inflammation propagation in ARDS (66).

Most recently, the changing epidemiology of AKI among the pandemic SARS-CoV-2 related ARDS cases worldwide has further highlighted the potential mechanistic link between a therapeutic approach and remote organ injury propagation. Many centers have reported a high incidence of renal replacement therapy requiring AKI in the setting of COVID-19 early on in the pandemic with dismal outcomes (81). The use of higher end pressures has been associated with AKI (62). Others have proposed that aggressive fluid restriction and attempt at decongestion through liberal diuretic use has exacerbated kidney injury and delayed renal recovery, leading to worsening of AKI and earlier use of RRT (82). The high incidence of AKI in patients with COVID-19 underscores dynamic interplay between the kidneys and the lungs.

Although our understanding remains incomplete, awareness of continuous interaction and organ crosstalk highlights the importance of vigilance related to lung protective ventilation strategies in the management of ARDS in order to limit lung mediated remote organ injury, such as AKI. In addition to inflammatory consequences, mechanical ventilation has a number of hemodynamic effects and influences neurohormonal factors that contribute to AKI. Elevated pulmonary vascular resistance and right ventricular strain in the setting of ARDS might impact renal perfusion (74). Elevated filling pressures contribute to decreased renal perfusion, especially in the setting of marginal arterial pressures, which can initiate and propagate AKI. In addition, mechanical ventilation can alter renal hemodynamics by increasing sympathetic tone and renin-angiotensin aldosterone system activation (83–85).

### Fluid Overload, ARDS Phenotypes and AKI

Knowledge of the pathophysiologic links between AKI and ARDS could be leveraged to identify patients with AKI who are at risk of developing ARDS and can be used to develop targeted therapies that prevent or treat this complication. Unfortunately, the complex interplay between both organ systems discussed thus far makes this endeavor less attainable in clinical practice. Lung protective mechanical ventilation and fluid management to restore effective arterial blood volume are the cornerstones of ARDS management. Currently, avoiding positive fluid balance remains the most important predictor of pulmonary complications in patients with AKI and is the primary target for intervention. Restrictive fluid management in ARDS has indeed become the standard of care (86, 87). Despite advances in medical care, treatment options remain limited and are largely supportive. Lack of positive data from multiple interventional randomized control trials has incited a search towards discerning molecular sub-phenotypes of ARDS based on clinical and biological determinants. Recent work aimed at understanding the different presentations clustered under the rather heterogeneous complex syndromic designation of ARDS has provided fascinating insight regarding patient and disease specific characteristics of ARDS and differential response to treatment that drive this complex construct.

Calfee et al. have described two different subphenotypes ARDS in patient cohorts enrolled in two randomized controlled trials based on clinical and biomarker variables derived from latent class analysis (1, 67). Importantly, both subphenotype classifications relied on the plasma biomarkers not available in routine clinical practice to delineate the two groups, signifying that these specific biomarkers may be uncovering aspects of underlying pathophysiology not captured by routine clinical variables. In these analyses, the inflammatory subphenotype 2 had higher levels of proinflammatory biomarkers such as IL-8, IL-6, and sTNF receptor 1, and higher plasma levels of and RAGE compared to subphenotype 1. Subjects with sepsis associated ARDS were more likely to belong to subphenotype 2 compared to subjects with trauma associated ARDS who were classified as subphenotype 1. Subjects with phenotype 2 were more likely to have higher mortality, and fewer organ failure free and ventilator free days. Most surprisingly, the two groups had differential response to treatment strategies: subjects in subphenotype 1 had higher mortality with higher versus lower PEEP; conversely, in subphenotype 2 higher PEEP was associated with lower mortality. Even more pertinent to our topic, the two subphenotypes had a differential response to fluid exposure; subphenotype 2 had a higher mortality when assigned to liberal fluid management strategy. Conversely, subphenotype 1 had a higher mortality with restrictive fluid management. The underlying reason for this differential response remains speculative. However, higher Ang-2 levels in subphenotype 2 signal endothelial damage, putting patients at risk of altered fluid excretion and fluid imbalance leading to fluid overload and higher mortality. Conversely, fluid restriction in patients sub phenotype 1 with lower Ang-2 levels could signify lower effective arterial blood volume and impaired end organ perfusion and higher mortality (5). Although subjects in subphenotype 2 had fewer ventilator free days compared to subphenotype 1, there was no association with fluid management strategy, perhaps signaling the contribution of other extra pulmonary organ injury such as AKI leading to fluid overload and worse pulmonary compliance. Interestingly, none of the extensive clinical variables tested was predictive of a subphenotype, suggesting that targeting clinically relevant subphenotypes might be the next strategy in designing interventional trials.

It is easy to speculate that pediatric AKI could very well represent a heterogenous syndrome with varied responses to fluid exposure significantly impacting lung-kidney interactions. Stratified analysis of a prospective study showed that mortality was associated with greater cumulative fluid balance on Day 3 of ARDS with concomitant AKI (88). In addition, higher degrees of inflammation, indicated by elevated IL-6 levels on day 1 were associated with positive cumulative fluid balance, AKI and, hence, mortality. Conceivably, higher IL-6 levels could signal a hyperinflammatory subphenotype of ARDS that might result in increased vascular permeability, endothelial damage, and AKI. Currently available data do not differentiate between adverse effects noted on the two subphenotypes of ARDS and the concomitant or sequential occurrence of oliguric vs. non-oliguric AKI. Oliguric AKI is known to carry a worse prognosis compared to non-oliguric AKI, yet available pediatric data are conflicting regarding oliguric AKI as a precursor to development of fluid overload (14, 89–93). Higher levels of inflammatory and endothelial activation mediators in the inflammatory subphenotype could result in microcirculatory dysfunction and energy failure leading to impaired fluid excretion, contributing to fluid overload and, hence, worse pulmonary compliance. Currently available adult data revealing the paradoxical response to fluid exposure suggest that the hyperinflammatory subphenotype could be the target population to test whether restrictive fluid strategy would help mitigate the effects of endothelial damage and vascular permeability, and related higher risk of fluid overload and AKI in this population. To help advance our understanding of the complex mechanisms involved in ARDS, AKI, and their interactions, it is crucial to identify if similar subphenotypes also exist in pediatric ARDS in order to develop and test an individualized approach to clinical management, specifically pertaining to ventilator and fluid exposure, to care for our patients with a more informed and personalized approach.

## Conclusions and Future Research Directions

Critical illness is largely a state of organ crosstalk and interaction; therefore, successful management requires a thorough understanding of its management. Changing epidemiology of pediatric AKI has clearly placed this syndrome in the setting of multiple organ dysfunction especially as it relates to the most severe forms of AKI with the poorest outcomes. Similarly, refinement of the pediatric ARDS definition has improved our understanding of the disease pathophysiology. As such, pediatric AKI and ARDS are the two most common organ failures intensivists deal with on a daily basis. Recent evidence, especially around mechanistic pathways in each syndrome, has enhanced our appreciation of the actual scale of organ crosstalk that extends beyond simply fluid accumulation and its management in AKI and ARDS. Multiple disease-specific molecular mediators and inflammatory pathways are involved in organ crosstalk in the AKI-ARDS construct, and the reciprocal hemodynamic effects of elevated pulmonary vascular resistance and central venous pressure (CVP) augment both renal and pulmonary congestion and impair renal oxygenation. Future research (Table 3) should be directed at further elucidating the molecular and cellular basis of kidney lung crosstalk, which will require the development of alternatives to animal models. Potentials strategies include systems biology applications such as “–omics” platforms and single cell sequencing of circulating immune cells, as well as the development of microfluidic organ-on-a-chip models that incorporate lung and kidney organoids to model organ crosstalk. Knowledge gained from these studies could be used to develop targeted therapies and molecular phenotyping tools that identify discrete subtypes of pediatric AKI and ARDS. Ultimately, this knowledge could arm clinicians with the tools for a precision medicine based approach, such as biomarker directed fluid management and other therapies. While it will be some time before these ambitious goals are realized, in the interim, clinicians must develop an appreciation of complexity of organ interactions and maintain vigilance regarding bidirectionality while treating these interrelated conditions in the critically ill pediatric patient.

**Table 3:** Future basic and translational research directions in kidney-lung crosstalk.

• Further elucidation of the molecular and cellular basis of organ crosstalk • Determination of ventilator and hemodynamic parameters that optimize renal perfusion in patients with ARDS • Development of alternatives to animal models for studying organ crosstalk, such as organoid-based microfluidic chips • Application of systems biology tools such as transcriptomics, proteomics, metabolomics, and single cell sequencing to identify molecular phenotypes of pediatric AKI and ARDS • Incorporation of molecular phenotyping into future clinical trial design

## Author Contributions

All authors listed have made a substantial, direct, and intellectual contribution to the work and approved it for publication.

## Conflict of Interest

The authors declare that the research was conducted in the absence of any commercial or financial relationships that could be construed as a potential conflict of interest.

## Publisher's Note

All claims expressed in this article are solely those of the authors and do not necessarily represent those of their affiliated organizations, or those of the publisher, the editors and the reviewers. Any product that may be evaluated in this article, or claim that may be made by its manufacturer, is not guaranteed or endorsed by the publisher.
